# Template-Free Synthesis of Magnetic La-Mn-Fe Tri-Metal Oxide Nanofibers for Efficient Fluoride Remediation: Kinetics, Isotherms, Thermodynamics and Reusability

**DOI:** 10.3390/polym14245417

**Published:** 2022-12-11

**Authors:** Shaoju Jian, Yuhuang Chen, Fengshuo Shi, Yifei Liu, Wenlong Jiang, Jiapeng Hu, Xiaoshuai Han, Shaohua Jiang, Weisen Yang

**Affiliations:** 1 Fujian Key Laboratory of Eco-Industrial Green Technology, Key Laboratory of Green Chemical Technology of Fujian Province University, College of Ecology and Resources Engineering, Wuyi University, Wuyishan 354300, China; 2 Jiangsu Co-Innovation Center of Efficient Processing and Utilization of Forest Resources, International Innovation Center for Forest Chemicals and Materials, College of Materials Science and Engineering, Nanjing Forestry University, Nanjing 210037, China

**Keywords:** La-Mn-Fe tri-metal oxides nanofibers, defluoridation, electrospinning, kinetics, wastewater treatment

## Abstract

The occurrence of fluoride contamination in drinking water has gained substantial concern owing to its serious threat to human health. Traditional adsorbents have shortcomings such as low adsorption capacity and poor selectivity, so it is urgent to develop new adsorbents with high adsorption capacity, renewable and no secondary pollution. In this work, magnetic electrospun La-Mn-Fe tri-metal oxide nanofibers (LMF NFs) for fluoride recovery were developed via electrospinning and heat treatment, and its defluoridation property was evaluated in batch trials. Modern analytical tools (SEM, BET, XRD, FTIR) were adopted to characterize the properties of the optimized adsorbent, i.e., LMF11 NFs with a La:Mn molar ratio of 1:1. The surface area calculated via BET method and pH_pzc_ assessed using pH drift method of LMF11 NFs were 55.81 m^2^ g^−1^ and 6.47, respectively. The results indicated that the adsorption amount was highly dependent on the pH of the solution, and reached the highest value at pH = 3. The kinetic behavior of defluoridation on LMF11 NFs was dominated by the PSO model with the highest fitted determination coefficients of 0.9999. Compared with the other three isotherm models, the Langmuir model described defluoridation characteristics well with larger correlation coefficients of 0.9997, 0.9990, 0.9987 and 0.9976 at 15 °C, 25 °C, 35 °C and 45 °C, respectively. The optimized LMF11 NFs exhibited superior monolayer defluoridation capacities for 173.30–199.60 mg F^−^/g at pH 3 at 15–45 °C according to the Langmuir isotherm model. A thermodynamic study proved that the defluoridation by LMF11 NFs is a spontaneous, endothermic along with entropy increase process. In addition, the LMF11 NFs still showed high defluoridation performance after three reused cycles. These findings unveil that the synthesized LMF11 NFs adsorbent is a good adsorbent for fluoride remediation from wastewater owing to its low cost, high defluoridation performance and easy operation.

## 1. Introduction

Fluoride contamination in groundwater caused by the processes of natural geochemical and human production activities is a burning issue, which has attracted substantial attention since it is closely related to the safety and health issue for humans [1,2,3,4,5,6]. An appropriate amount of F^−^ can enhance the hardness of teeth and the mineralization of hard tissues and prevent dental caries. Surplus fluoride in drinking water may lead to irremediable diseases in humans, namely fluorosis. The tolerance level of fluoride in drinking water recommended by the World Health Organization is no more than 1.5 mg/L [7,8]. However, endemic levels of fluorosis have been reported in 32 nations worldwide, and more than 200 million people especially in poor rural areas have been consuming water contaminated with high fluoride concentration for a long time. Accordingly, it is highly required to establish the deep treatment of fluoride-rich water. Adsorption is a promising way for fluoride decontamination since it possesses high adsorption capacity, high selectivity, handy operation and relative cost-effectiveness [9,10,11,12,13,14], compared with other defluoridation strategies, e.g., membrane, precipitation, and electrocoagulation [15]. A variety of defluoridation materials have been developed to remove excess F^−^, namely carbon-based materials [16], activated alumina [17], carbonaceous materials [18], polymer-based materials [19], etc. Whereas the inherent shortcomings such as poor removal efficiency and low selectivity, have been encountered in practical applications for most of these adsorbents.

As far as we know, Belcher R. [20,21] in 1959 and Leonard et al. [22] in 1960 discovered for the first time that the coordination water in the complexes formed by rare earth elements Ce(III) and La(III) with alizarin complexone could exchange with F^−^ to form colored complexes with high selectivity, respectively. It provided a new way for scientists to remove fluoride from wastewater. Since then, various materials, such as compounds of multivalent metal elements such as La(III), Ce(III) and Ce(IV) in the form of oxides, hydrous oxides and basic carbonate [23], lanthanum impregnate silica gel [24], La(III) and Y(III)-impregnated alumina [25], La-chitosan [26], Mn-La metal composite [27], Fe-La-Ce tri-metallic composite [28], La-MOFs [29], La-MOF@x%PANI [30], had been exploited for remediation of fluoride. Among them, bi-metallic or tri-metallic oxides based on high valent lanthanum possessed high fluoride adsorption. However, the cost of La is relatively high. In order to reduce the cost and keep high defluoridation performance, adsorbents for fluoride remediation were fabricated by mixing other cheaper multivalent metal elements (i.e., Mg, Al, Mn, Fe) with La owing to the advantage and synergism of two or three metal oxides [31,32,33,34]. Mg-Fe-La tri-metal adsorbent (molar ratio: 25/1/4) developed by Wang et al. [32] via facile co-precipitation method possessed the highest uptake of 112.17 mg F^−^/g for fluoride. Cotton-like Ca-Al-La adsorbent with optimal Ca/Al/La molar ratio of 1/4/2 exhibited the largest fluoride binding amount of 29.30 mg F^−^/g [35]. Similarly, Gasparotto et al., stated that La-Al-Fe trioxide composites synthesized via the co-precipitation method presented a maximum fluoride uptake of 28.06 mg F^−^/g at 25 °C and pH 8.25 [36]. Although the above adsorbents possess good defluoridation performance, it is difficult to separate them from aqueous solution quickly due to their nanoscale size. Additionally, the nano-adsorbents are easy to agglomerate, resulting in the decline of adsorption capacity. 

Ease in localization and separation can promote the recovery and reuse of materials for successive treatment cycles. Due to the good magnetic responsiveness of Fe_3_O_4_ [37,38], its use in composite adsorbents for various pollutants removal can enhance their recovery rate and efficiency by a magnet [39,40]. Whereas, easy agglomeration is the major obstacle for the large-scale application of Fe_3_O_4_ nanoparticles. To overcome the limit of Fe_3_O_4_ nanoparticles, numerous magnetic adsorbents were fabricated by coating metal oxides on Fe_3_O_4_ nanoparticles to form core-shell composites or by loading Fe_3_O_4_ and other metal oxides on the carrier [41]. For example, graphene oxide (GO) supported Fe-Al oxide adsorbent (IAO/GO) fabricated by Liu et al. [42] showed a maximum binding amount of 64.72 mg F^−^/g for fluoride. The use of magnetic La_2_O_3_-CeO_2_-Fe_3_O_4_ nanofibers for fluoride removal from aqueous solution has been investigated by our group [43], the maximum uptakes are 202–230 mg F^−^/g at 15–35 °C, and the adsorbent can be easily and quickly separated from the solution by an external magnet. The obstacles to the industrial application of La_2_O_3_-CeO_2_-Fe_3_O_4_ nanofibers are the scarcity and relatively high cost of rare earth elements. There is no literature about the defluoridation by magnetically separable electrospun La-Mn-Fe tri-metal oxide nanofibers in wastewater. In view of the high affinity of La towards fluoride ions and the low cost of metals Mn and Fe, this research is to fabricate a new magnetic tri-metallic adsorbent (i.e., electrospun La-Mn-Fe tri-metal oxide nanofibers) with less expenditure, which can exhibit structural stability, high defluoridation efficiency, excellent recyclability and reusability.

In this study, magnetically separable electrospun La-Mn-Fe tri-metal oxide nanofibers (LMF NFs) were prepared by electrospinning and heat treatment. The LMF NFs were used as a new type of adsorbent for fluoride remediation. The magnetic Fe_3_O_4_ NPs are evenly dispersed along the nanofiber axis without obvious agglomeration. Moreover, the magnetic fibrous LMF NFs can effectively prevent the agglomeration of adsorbent during the defluoridation process and can be quickly separated from the solution using an external magnet after adsorption. The effects of the molar ratio of La/Mn, initial F^−^ concentration, pH, LMF11 NFs dosage, shaking time, interfering anions, isotherm models and adsorption kinetics were investigated in detail.

## 2. Materials and Methods

### 2.1. Chemicals

NaF, La(NO_3_)_3_·6H_2_O, Mn(CH_3_COO)_2_·4H_2_O, NaCl, DMF, Na_2_CO_3_, NaNO_3_, Na_3_PO_4_, CH_3_COOH, Na_3_C_6_H_5_O_7_·2H_2_O, Na_2_SO_4_, HCl, NaOH, FeCl_3_·6H_2_O, HOCH_2_CH_2_OH and CH_3_COONa were acquired from Aladdin Chemical Reagent Co., Ltd. (Shanghai, China) and used as received. Polyacrylonitrile (PAN, Mn = 2.5 × 10^5^) was purchased from Sigma-Aldrich (St. Louis, MO, USA).

### 2.2. Preparation of La-Mn-Fe Tri-Metal Oxide Nanofibers

**Scheme 1:**
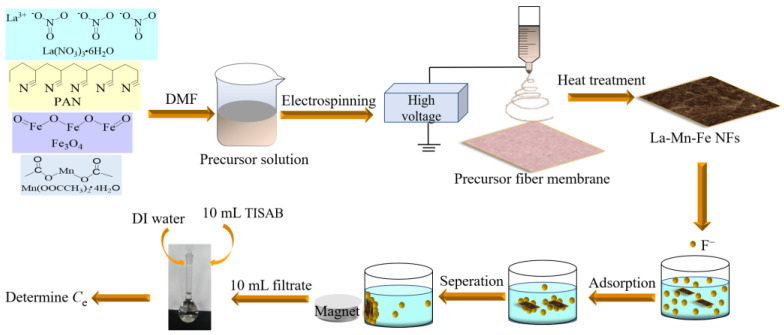
Graphical procedure of fabrication of LMF NFs and the adsorption of fluoride.

La(NO_3_)_3_·6H_2_O and Mn(CH_3_COO)_2_·4H_2_O were mixed in a beaker with La:Mn molar ratios of 2:1, 1:1 and 1:2, then completely dissolved in a small amount of DMF. Afterward, 1.8 g PAN powder, 10 mL DMF and 0.075 g Fe_3_O_4_ nanoparticles (via a solvothermal method [44]) were injected into each sample to form precursor solutions namely (La(NO_3_)_3_/Mn(CH_3_COO)_2_/Fe_3_O_4_/PAN/DMF). The electrospinning processes were conducted to produce precursor fibers [33,43,45,46]. Briefly, the precursor solution was added into a 5 mL syringe with a metal needle, then the electrospinning of the precursor solution was performed with an applied electric field of 150 kV m^−1^ and a flow rate of 1.2 mL h^−1^ at room temperature, to generate precursor nanofibers. After electrospinning, the resulting precursor nanofibers were dehydrated for 8 h and then put into a tube furnace for heat treatment at 500 °C for 2 h to prepare La-Mn-Fe tri-metal oxide nanofibers under an air atmosphere. La-Mn-Fe tri-metal oxide nanofibers with molar ratios of La/Mn being 2/1, 1/1 and ½ were noted as LMF21 NFs, LMF11 NFs and LMF12 NFs, respectively. The graphic plan of the fabrication process of electrospun LMF NFs and the adsorption of fluoride is portrayed in Scheme 1.

### 2.3. Batch Adsorption Studies

The defluoridation performance of LMF NFs was evaluated using batch experiments (Scheme 1). Adsorption kinetics were performed in the time range from 1 to 60 min under 500 mL F^−^ solution with initial F^−^ concentrations of 20 and 50 mg F^−^/L at 25 °C and pH 3. Adsorption isotherm studies were carried out at varying original F^−^ concentrations of 10–60 mg F^−^/L at a temperature range of 15–45 °C with LMF11 NFs dosage of 0.2 g/L in 50 mL of respective solution. Various concentrations (10, 30, 50, 80 or 100 mg/L) of five commonly occurring anions, including nitrate (NO_3_^−^), sulfate (SO_4_^2−^), chloride (Cl^−^), carbonate (CO_3_^2−^), phosphate (PO_4_^3−^) were studied under initial F^−^ concentration of 20 mg F^−^/L with an adsorbent dosage of 0.2 g/L at 25 °C and pH 3 for 12 h. The effect of pH (2–9) along with LMF11 NFs dosage (0.2–0.6 g/L) on adsorption property was assessed under 50 mL initial F^−^ concentration of 20 mg F^−^/L at 25 °C for 12 h.

### 2.4. Reusability Test

The reusability of the F^−^ adsorbed magnetic LMF11 NFs was determined to quantify the cost-effectiveness of the adsorbent for F^−^ remediation in the successive adsorption–desorption cycles. The original concentration of F^−^ was 20 mg F^−^/L at pH 3 and 25 °C for 8 h for each regeneration cycle. The F^−^ adsorbed magnetic LMF11 NFs were recovered by an external magnet, and then eluted with 100 mL of 0.1 M NaOH to regeneration [43,47,48], vacuum-dried and used for a new run.

### 2.5. Characterization of Adsorbent

The crystal structures of LMF11 NFs before and after adsorption were established by X-ray diffraction (XRD, D8 ADVANCE X, Bruker, Saarbrucken, Germany), The BET specific surface area of the LMF11 NFs adsorbent was examined using a Micrometrics ASAP 2020 analyzer (Micromeritics, Norcross, GA, USA). The TEM and SEM images of the magnetic electrospun LMF11 NFs were acquired from TEM JEM 2100 (JEOL, Tokyo, Japan) and SEM 300U (VEGA, Tescan, Brno, Czech Republic). The point of zero charge of LMF11 NFs was assessed via a pH drift method [49].

## 3. Result and Discussion

### 3.1. Characterization of Adsorbent

**Figure 1:**
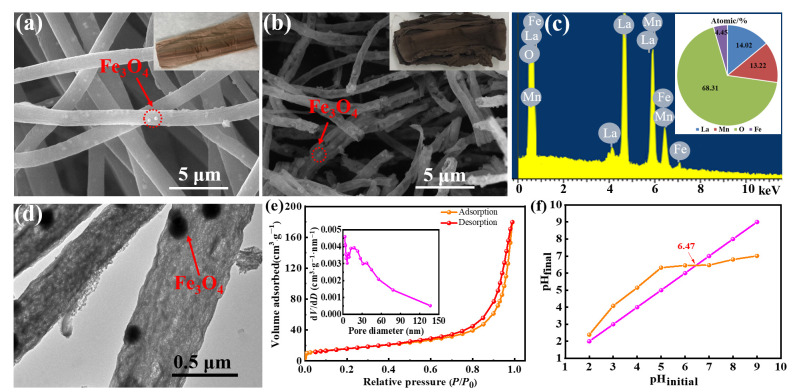
SEM images of La(NO_3_)_3_/Mn(CH_3_COO)_2_/Fe_3_O_4_/PAN (**a**) and LMF11 NFs (**b**), EDS analysis (**c**), TEM image (**d**), N_2_ adsorption–desorption isotherm (**e**) and (**f**) pH_pzc_ of LMF11 NFs.

The morphological details of La(NO_3_)_3_/Mn(CH_3_COO)_2_/Fe_3_O_4_/PAN and LMF11 NFs were determined by SEM, as portrayed in Figure 1a,b. Obviously, the surfaces of La(NO_3_)_3_/Mn(CH_3_COO)_2_/Fe_3_O_4_/PAN fibers are continuous and smooth with an average diameter of 1.28 ± 0.10 μm. Fe_3_O_4_ NPs are evenly distributed on the surface of the La(NO_3_)_3_/Mn(CH_3_COO)_2_/Fe_3_O_4_/PAN and LMF11 NFs. After heat treatment, the volume and diameter of fibers decline due to the decomposition of Mn(CH_3_COO)_2_, La(NO_3_)_3_ and PAN. The continuous LMF11 NFs are hollow and rougher compared to that before calcination, which possess an average diameter of 626 ± 57 nm. Figure 1d displays the TEM image of LMF11 NFs, it is obvious that the spherical Fe_3_O_4_ NPs are uniformly dispersed along the fiber axis without obvious agglomeration. The chemical composition of LMF11 NFs is verified by EDS analysis, as shown in Figure 1c. EDS illustrates that the LMF11 NFs consist of La (14.02%), Mn (13.22%), Fe (4.45%) and O (68.31%) elements. Apparently, the detected La/Mn molar ratio in the sample is quite consistent with the designed value. The BET surface area and BJH pore distribution of LMF11 NFs are analyzed by N_2_ adsorption–desorption isotherm (Figure 1e). The total pore volume and surface area of LMF11 NFs are 0.276 cm^3^ g^−1^ and 55.81 m^2^ g^−1^. The inset of Figure 1e states that the main pore sizes of LMF11 NFs locate at 3.06 nm and 17.39 nm, indicative of numerous mesopores in the adsorbent [29]. The isoelectric point (pH_pzc_) of LMF11 NFs calculated according to the literature [50] is 6.47 (Figure 1f). This signifies that the surface charge of LMF11 NFs is positive and protonated at pH < 6.47 while negative at pH > 6.47. Thus, negative ions such as F^−^ can be could easily absorbed into the positively charged LMF11 NFs owing to their columbic attractions at a pH range below pH_pzc_ [51]. 

### 3.2. Preparation Optimization

**Figure 2:**
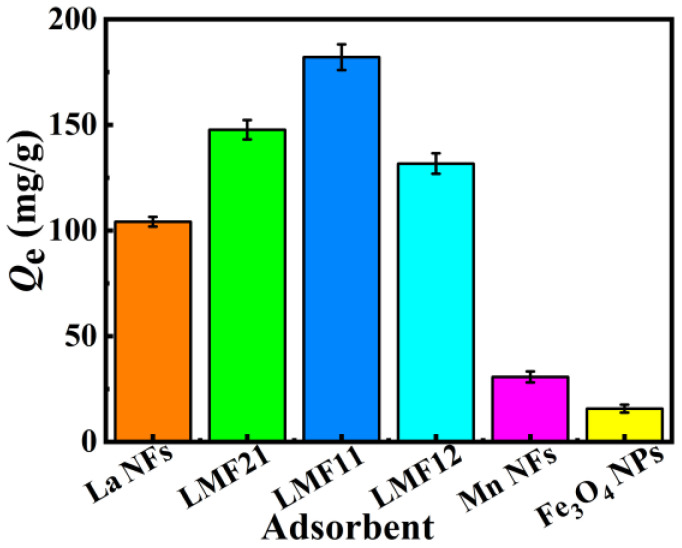
Impact of La/Mn molar ratio on the defluoridation performance of LMF NFs.

The capture capacities of F^−^ on as-prepared adsorbents involving La NFs, LMF21 NFs, LMF11 NFs, LMF12 NFs, Mn NFs and Fe_3_O_4_ NPs were determined. Then, 0.2 g/L of adsorbents were placed into 50 mL fluoride solutions with a concentration of 20 mg F^−^/L for the adsorption test, the temperature of 25 °C, pH of 3 and shaking time for 12 h were adopted, respectively. The fluoride binding amounts were determined, as portrayed in Figure 2. The maximum fluoride uptakes of La NFs, Mn NFs and Fe_3_O_4_ NPs are 104.12, 30.71 and 15.71 mg F^−^/g, respectively. While the maximum uptakes of F^−^ on LMF21 NFs, LMF11 NFs, LMF12 NFs are 147.68, 182.03 and 131.67 mg F^−^/g, respectively. Evidently, the LMF NFs possess higher adsorption capacity than those of single metal oxide due to the synergistic effect of La, Mn and Fe [33,34]. The fluoride uptake of LMF11 adsorbent with La/Mn molar ratio being 1/1 is the highest. Hence, LMF11 NFs is designed as fluoride scavenger in the subsequent experiments.

### 3.3. Adsorption Study

#### 3.3.1. The Effect of pH

**Figure 3:**
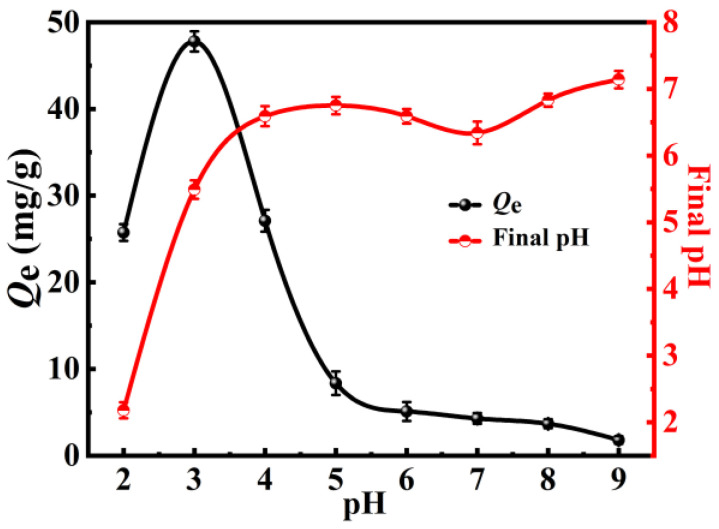
pH effect on defluoridation performance on LMF11 NFs and the final pH of the solution.

The effect of pH on the defluoridation performance of LMF11 NFs was evaluated at various pH values from 2 to 9 (Figure 3). The maximum binding amount (47.79 mg F^−^/g) and removal efficiency (95.58%) for LMF11 NFs occurs at a pH of 3 owing to the columbic attractions (Equation (1)). LMF11 NFs show a dramatic decrease in defluoridation property at pH > 3 and pH < 3. Most fluoride ions exist in the form of electrically neutral HF (pKa^HF^ = 2.95) [52] when pH < 3, which can reduce the columbic forces between LMF11 NFs and adsorbate [42]. Meanwhile, the diminished binding amount at higher pH conditions is attributed to electrostatic repulsion between F^−^ and negatively charged LMF11 NFs along with the competition between F^−^ and OH^−^ for active sites [41]. Additionally, the equilibrium pH of the F^−^ solution changed from 2 to 2.18, 3−5.49, 4–6.59, 5–6.75, 6–6.59, 7–6.34, 8–6.83, 9–7.14 after adsorption, respectively. The improvement of pH after adsorption in an initial pH range of 2–6 suggests the existence of an ion exchange of hydroxyl groups bonded on the surface of the adsorbent with fluoride ions (Equations (2) and (3)). While the decline of pH after adsorption in an initial pH range from 7 to 9 (>pH_pzc_) is mainly ascribed to the competitive adsorption between hydroxyl groups and adsorbate (F^−^) (Equation (4)). A pH of 3 was chosen to utilize for subsequent studies.

≡M–OH_2_^+^ + F^−^ → ≡MOH_2_^+^–F^−^ (s) + H_2_O (1)

≡M–OH(s) + H_2_O + F^−^ → ≡MOH_2_^+^–F^−^ (s) + OH^−^ (2)

≡M–OH(s) + F^−^ → ≡M–F(s) + OH^−^ (3)

≡M–OH + F^−^+ OH^−^ → M–O^−^ + F^−^+ H_2_O (4)

where ≡M indicative of La, Fe and Mn metal ions.

#### 3.3.2. The Effect of LMF11 NFs Dosage

**Figure 4:**
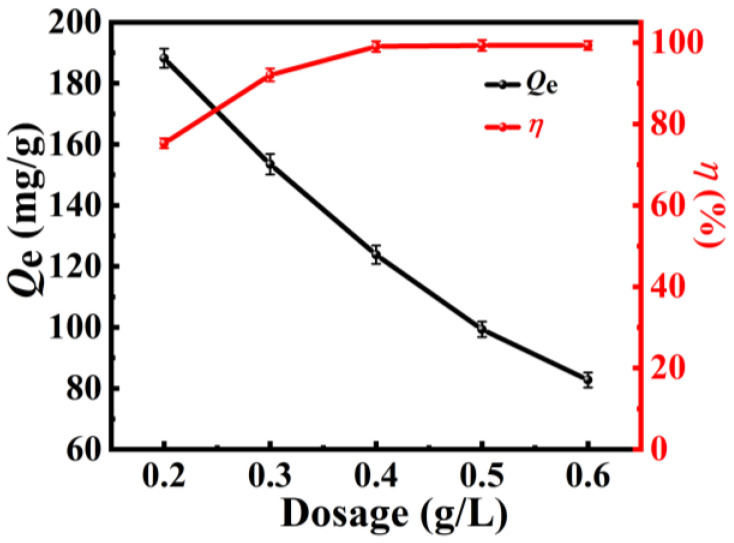
Effect of adsorbent dosage on the defluoridation performance of LMF11 NFs.

The effect of LMF11 NFs dosage on the defluoridation was studied. Appropriate mass (0.01, 0.015, 0.02, 0.025, or 0.03 g) of LMF11 NFs was poured into a 50 mL of 50 mg F^−^/L fluoride solution at 25 °C. Evidently, the adsorption uptakes at an adsorbent dosage of 0.2, 0.3, 0.4, 0.5 and 0.6 g L^−1^ are 188.21, 153.44, 123.81, 99.33 and 82.77 mg F^−^/g, the fluoride removal percentages are 75.29%, 92.07%, 99.05%, 99.32% and 99.33%, respectively (Figure 4). It can be concluded that the binding amount descends progressively with ascending LMF11 NFs dosage at a fixed concentration of F^−^; the removal efficiency shows is an increasing trend. The number of the binding sites on LMF11 NFs rises with the increase in the LMF11 NFs dosage, the solution, resulting in an obvious increase in fluoride removal percentage. However, the initial concentration of F^−^ keeps constant and fails to saturate the binding sites on LMF11 NFs. It means that the binding sites cannot be fully used. In addition, the binding sites may be agglomerated together, leading to partial binding sites covering each other, and then the unit adsorption capacity cuts down [41]. The adsorbent dosage is selected as 0.2 g/L in the following defluoridation experiments in light of the economy and practicability of the adsorbent.

#### 3.3.3. The Effect of Initial F^−^ Concentration (C_0_)

**Figure 5:**
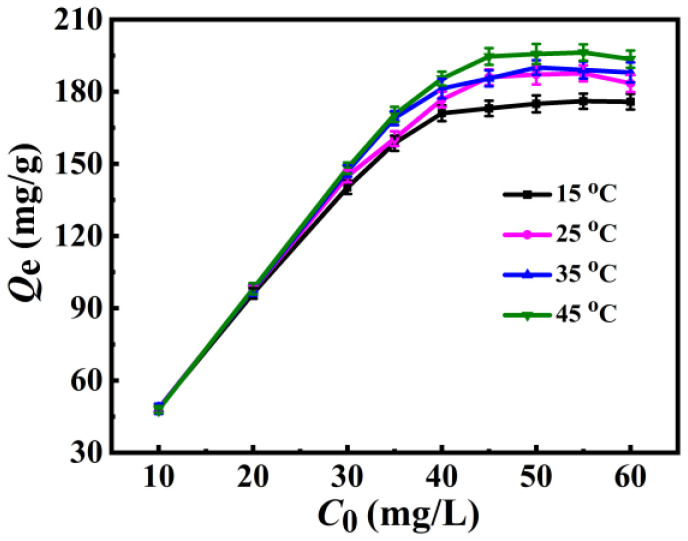
Influence of initial concentration of F^−^ on the defluoridation performance of LMF11 NFs.

The fluoride uptake significantly depends on the initial concentration of F^−^. As shown in Figure 5, the fluoride uptake of LMF11 NFs elevates gradually with an increase in *C*_0_ (10 mg F^−^/L to 45 mg F^−^/L). The reason is that with the increase in F^−^ concentration, the number of fluoride ions near the surface of the LMF11 NFs increases apparently, and the binding sites on the surface of LMF11 NFs are more fully surrounded by fluoride ions, thus, more fluoride ions are adsorbed by the adsorbent, resulting in the increasing of fluoride binding amount [53]. Then it approaches saturation at higher *C*_0_ due to the saturation of active sites. While the fluoride removal efficiency declines with the rise of fluoride concentration. It is because the LMF11 NFs adsorbent have a limited adsorption uptake. When LMF11 NFs adsorbent achieve adsorption saturation, the removal percentage will decline with the rise of the initial fluoride concentration. In addition, at *C*_0_ = 60 mg F^−^/L, the binding amount leaps from 175.82 mg F^−^/g at 15 °C to 193.53 mg F^−^/g at 45 °C. The higher the temperature of the adsorption system, the larger the binding amount, validating the endothermic nature of the defluoridation process [29]. 

#### 3.3.4. The Effect of Contact Time

**Figure 6:**
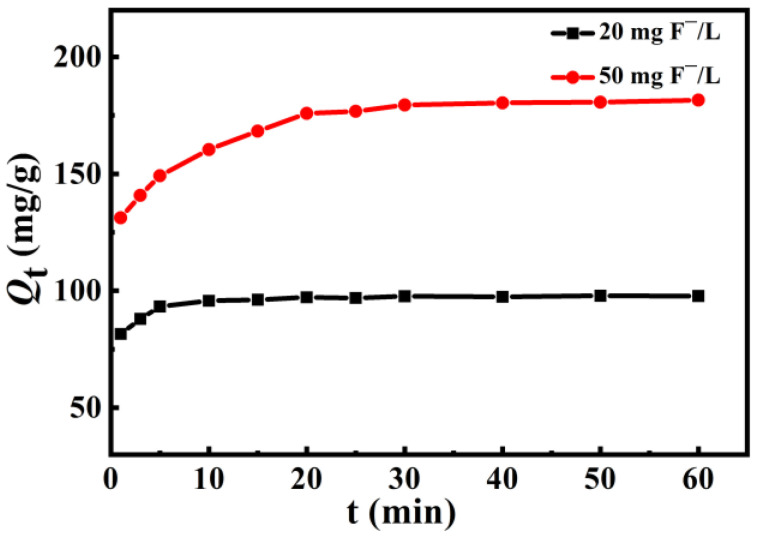
Influence of shaking time and initial F^−^ concentration on defluoridation performance.

The defluoridation experiments were kinetically conducted towards F^−^ removal on LMF11 NFs by varying the time intervals in the range of 0–60 min using an adsorbent dosage of 0.2 g/L with given concentrations (*C*_0_ = 20 and 50 mg F^−^/L) at 25 °C and pH = 3. As depicted in Figure 6, exceeding 70% of F^−^ could be removed within the first 20 min at *C*_0_ = 20 and 50 mg F^−^/L, showing an excellent defluoridation property. In this stage, numerous binding sites in LMF11 NFs and the high concentration of fluoride ions in the solution decide the rapid adsorption rate. Hence the larger adsorption rate in the incipient step is ascribed to the strengthening of the diffusion rate of F^−^ provided by concentration gradient along with the existence of numerous available binding sites on the LMF11 NFs [54]. The adsorption tends to dynamic equilibrium after 10 min in 20 mg F^−^/L solution and 30 min in 50 mg F^−^/L solution. Clearly, at higher *C*_0_, it takes longer shaking time to attain equilibrium. Based on the aforementioned analysis, the effect of pH (*A*), initial concentration of F^−^ (*B*) and LMF11 NFs dosage (*C*) (Supplementary Materials, Tables S1 and S2) was determined using response surface methodology (RSM) to optimize the adsorption parameters [55,56]. It is clear that the experimental results of the single factor variable are very close to the RSM results (Supplementary Materials, Table S3, Figures S1 and S2).

#### 3.3.5. The Effect of Interfering Anions

**Figure 7:**
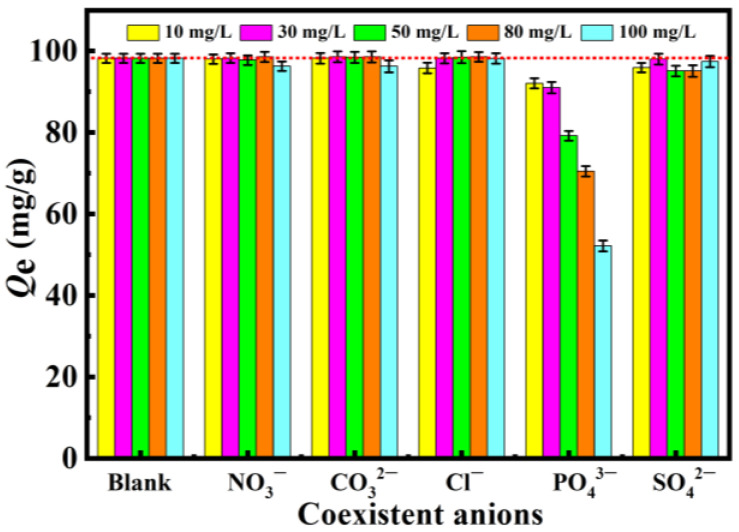
Effect of interfering anions on the fluoride adsorption density by using LMF11 NFs.

One of the major barriers that limit the widespread application of adsorption technology in practical water treatment is the selectivity of adsorbent. The effects of five commonly occurring anions including nitrate (NO_3_^−^), sulfate (SO_4_^2−^), chloride (Cl^−^), carbonate (CO_3_^2−^) and phosphate (PO_4_^3−^) on defluoridation by LMF11 NFs were investigated. The competitive experiments were conducted by weighing 0.01 g LMF11 NFs into a binary system that contained 50 mL 10 mg F^−^/L F^−^ paired with different concentrations (10, 30, 50, 80 or 100 mg/L) of the interfering anions, respectively. The result is depicted in Figure 7. Obviously, NO_3_^−^, Cl^−^, SO_4_^2−^ and CO_3_^2−^ anions at all concentrations of 10–100 mg/L almost do not prevent the F^−^ removal, illustrating the excellent selectivity and stability of LMF11 NFs adsorbent. Whereas the binding capacity drops quickly from 98.20 to 52.16 mg F^−^/g with the rise of PO_4_^3−^ concentration from 0 to 100 mg/L, manifesting a remarkable competitive effect of PO_4_^3−^ on defluoridation. This may be explained by the *K*_sp_ of LaPO_4_ (3.7 × 10^−23^) [54], which favors PO_4_^3−^ to displace the adsorbed F^−^ on LMF11 NFs compared with that of LaF_3_, thereby deteriorating the fluoride binding capacity. The same result has also been reported by several studies using rare earth element-based materials for fluoride remediation [27,57].

#### 3.3.6. Fluoride Adsorption Isotherm

**Figure 8:**
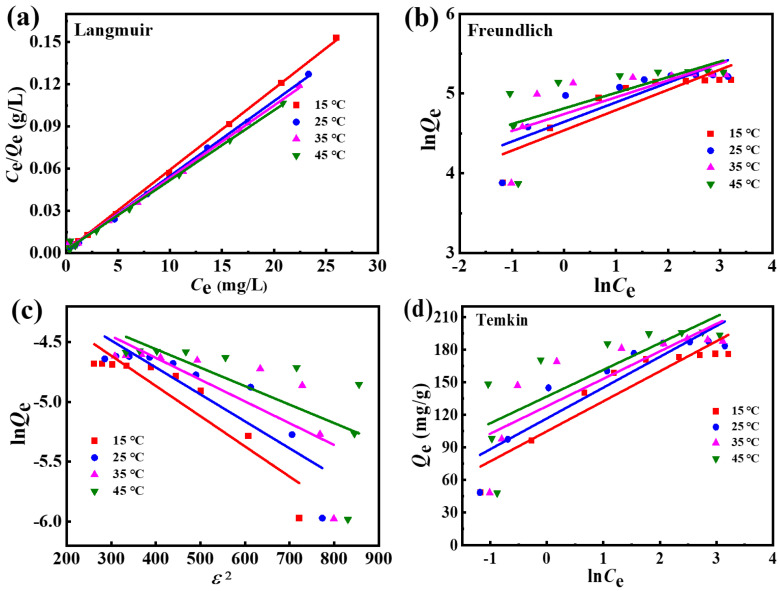
Adsorption isotherms of F^−^ on LMF11 NFs, (**a**) Langmuir, (**b**) Freundlich, (**c**) D–R and (**d**) Temkin at 15–45 °C.

The equilibrium data generated from the adsorption experiment at temperatures of 15 to 45 °C (Figure 5) are fitted by four classical isotherm modes viz., Langmuir, Freundlich, D–R and Temkin [29]. The as-estimated relevant factors are listed in Table 1. As portrayed in Figure 8a–d, the order of the fitted determination coefficients (*R*^2^) at 15 to 45 °C is Langmuir (0.9997, 0.9990, 0.9987 and 0.9976) > Temkin (0.8708, 0.8358, 0.6854 and 0.6161) > D–R (0.8402, 0.7725, 0.5981 and 0.4976) > Freundlich (0.7863, 0.7252, 0.5627 and 0.4791), illustrating that the homogeneous and single-layer adsorbed on LMF11 NFs dominates the defluoridation process [29,58]. The evaluated *Q*_m_ values range from 173.30–199.60 mg F^−^/g at 15 to 45 °C, exceedingly higher than many reported adsorbents including MOFs such as La–BTC (105.2 mg/g), La–BPDC (125.9 mg/g), La–BHTA (145.5 mg/g), La–PMA (158.9 mg/g) and La–BDC (171.7 mg/g) [29], Uio–66 (20 mg/g) [59], Amine functionalized electrospun cellulose nanofibers (5.31 mg/g) [60], electrospun alumina nanofibers (1.2 mg/g) [61] and double (Ce–Fe bimetal oxides, 60.97 mg/g [62]) or tri-metal oxide-based materials (SA–CMAZ, 31.72 mg/g [63]; Mg/Ce/Mn, 12.99 mg/g [64]). Table 2 lists the fluoride adsorption capacities of the LMF11 NFs and other adsorbents based on rare earth elements for comparison. It is conspicuously seen that LMF11 NFs show excellent fluoride adsorption capacity. Additionally, the dimensionless constant (*R*_L_) > 1 means unfavorable adsorption, while *R*_L_ < 1 indicates favorable adsorption processes [65]. All the attained values of *R*_L_ at evaluated temperatures lie between 0 and 1 (Table 1), reflecting favorable adsorption by LMF11 NFs in this study. 

The values of 1/*n* (0.1961–0.2543) established from the Freundlich model lie in the range of 0 < 1/*n* < 1 at 15 to 45 °C, signifying beneficial adsorption by LMF11 NFs under studied conditions [52]. The *E* value derived from the D-R model represents free energy. *E* values in ranges of 1–8 kJ mol^−1^, 8–16 kJ mol^−1^ and greater than 16 kJ mol^−1^ are indicative of electrostatic physical adsorption, electrostatic interaction or synergy and chemical adsorption, respectively [65]. The *E* value calculated from the D-R model gradually elevates from 14.06 to 17.96 kJ/mol with ascending the temperature from 15 to 45 °C, which denotes that the defluoridation on LMF11 NFs proceeds from ion exchange to chemisorption [66]. 

**Table 2:** Comparison of various adsorbents based on rare earth elements for defluoridation.

Adsorbents	pH	*C*_0_ (mg L^−1^)	Equilibrium Time/(h)	*Q*_m_ (mg g^−1^)	References
Ce-AlOOH	3	10–1000	2	62.8	[67]
Fe-Mg-La tri-metal nanocomposite	7	10–300	4	47.20	[66]
Fe-Al-Ce tri-metal oxide	5.8	2–110	24	195	[68]
Mg-Fe-La tri-metal composite	neutral pH	10–150	6	112.17	[32]
Bx-Ce-La@500	3	10–50	3	88.13	[69]
Fe-La-Ce tri-metallic composite	4	10–100	2	303.03	[28]
Zr-La/PP	3	2–50	8.3	32.5	[70]
Layered Zr-Al-La tri-metal composite	3	20–200	6.6	90.48	[71]
Granular TiO_2_-La	3–9	0.5–2830	–	78.4	[72]
La/Mg/Si-loaded palm shell-based activated carbon	7	100	2	285.7	[73]
La-Mn bimetal oxide nanofibers	3	10–55	1.5	189.39	[33]
Powdered Ce-Mn oxide	6	10–100	3	137.5	[74]
La-Mn-Fe NFs	3	10–60	1	199.60	This study

#### 3.3.7. Adsorption Kinetic

**Figure 9:**
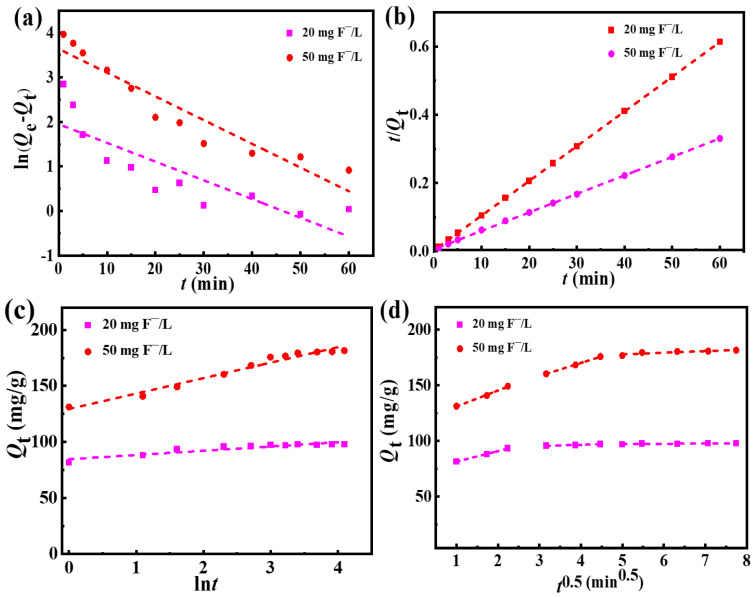
Linear kinetic models of F^−^ adsorption on LMF11 NFs, (**a**) PFO, (**b**) PSO, (**c**) Elovich and (**d**) W-M model.

Figure 9 depicts four linear adsorption kinetic curves namely PFO, PSO, Elovich and Weber and Morris models [53] of LMF11 NFs for fluoride at *C*_0_ = 20 and 50 mg F^−^/L and pH = 3. Correlative parameters are listed in Table 3. The highest regression coefficients (i.e., 0.9999 at 20 mg F^−^/L and 0.9999 at 50 mg F^−^/L) derived from the PSO model are larger than those of PFO (0.7212 and 0.9019) and Elovich (0.8714 and 0.9698) models, elucidating the chemisorption behavior plays a critical role in defluoridation process, which is consistent with the reported results of fluoride removal on other La-based adsorbents [32,75]. The theoretical fluoride capture capacities (*Q*_e, cal_, 98.23 and 184.50 mg F^−^/g at 20 and 50 mg F^−^/L) derived from the PSO model are in agreement with measured adsorption amounts (*Q*_e, exp_, 97.76 and 181.49 mg F^−^/g at 20 and 50 mg F^−^/L). The second-order rate constants (*k*_2_) are 3.7012 × 10^−2^ and 0.5236 × 10^−2^ g mg^−1^ min^−1^ at *C*_0_ = 20 and 50 mg F^−^/L and 25 °C, respectively. This indicates that LMF11 NFs exhibit a higher fluoride removal rate at a lower F^−^ concentration, resulting in a shorter time needed to attain the adsorption equilibrium. Similar results were observed in other La-modified adsorbents [54]. In addition, on the basis of the three-segment linear fitting Weber and Morris model, the intercepts (*C*) are not zero (Figure 9d), reflecting that the defluoridation mechanism of LMF11 NFs is complex and composed of surface adsorption, intraparticle diffusion along with external liquid film diffusion [48]. The order of *k*_int_ is *k*_int1_ > *k*_int2_ > *k*_int3_ at *C*_0_ = 20 and 50 mg F^−^/L, revealing that the surface or film diffusion is a rate-controlling step. The fluoride ions adsorb quickly on the external surface of LMF11 NFs at beginning. Pore diffusion or intraparticle is a rate-limiting step at the second stage and the diffusion into mesopores/micropores is dominant and then achieves equilibrium on the exterior surface at the third stage, where intraparticle diffusion decreases owing to the extremely low F^−^ concentration in the adsorption system [43,48].

#### 3.3.8. Thermodynamic Study

**Figure 10:**
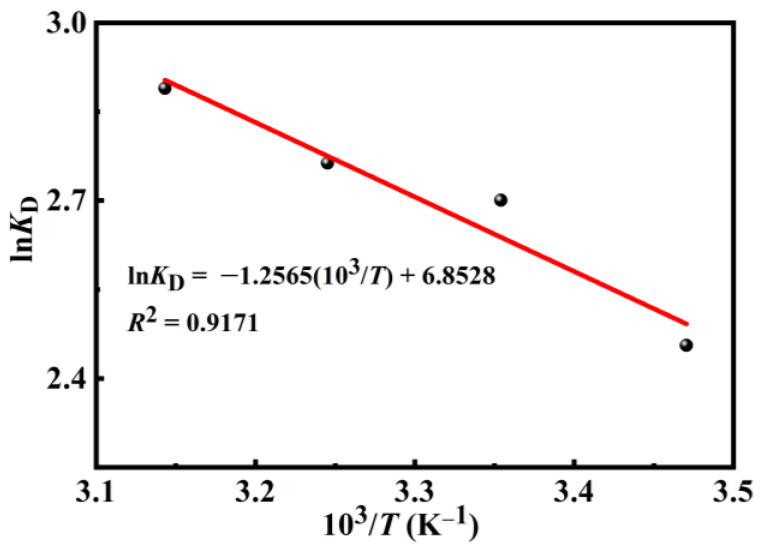
The linear relationship between ln*K*_D_ and 10^3^/*T*.

The thermodynamic factors (namely Δ*G*^0^, Δ*H*^0^ and Δ*S*^0^) derived from the plot of ln*K*_D_ versus 10^3^/*T* (Figure 10) are tabulated in Table 4. The Δ*G*^0^ values at 15–45 °C are assessed at −5.89, −6.69, −7.08 and −7.64 kJ mol^−1^, respectively. The values of Δ*G*^0^ are negative, signifying the defluoridation process by LMF11 NFs is feasible and spontaneous [75]. The positive Δ*H*^0^ (10.45 kJ mol^−1^) and Δ*S*^0^ (69.12 J mol^−1^ k^−1^) confirm the defluoridation by LMF11 NFs is endothermic along with an entropy increase process [76].

#### 3.3.9. Reusability

**Figure 11:**
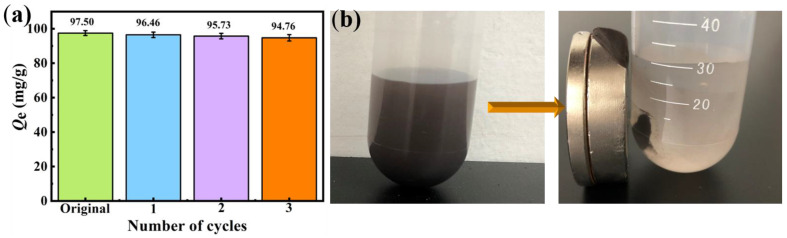
Reusability of LMF11 NFs (**a**) and photo of magnetic separation (**b**).

For an ideal adsorbent, it is vital to possess high reusability without a significant loss of removal efficiency. Figure 11 shows the renewability of LMF11 NFs. Notably, the F^−^ uptakes of LMF11 NFs are 97.50, 96.46, 95.73 and 94.76 mg F^−^/g at the original, first, second and third cycles, respectively. It can be concluded that the defluoridation performance is still effective for up to three consecutive reuse–regeneration cycles. The result states that LMF11 adsorbent exhibits sufficient chemical stability and reusability, thereby it is suitable for F^−^ removal from wastewater.

### 3.4. Adsorption Mechanism

**Figure 12:**
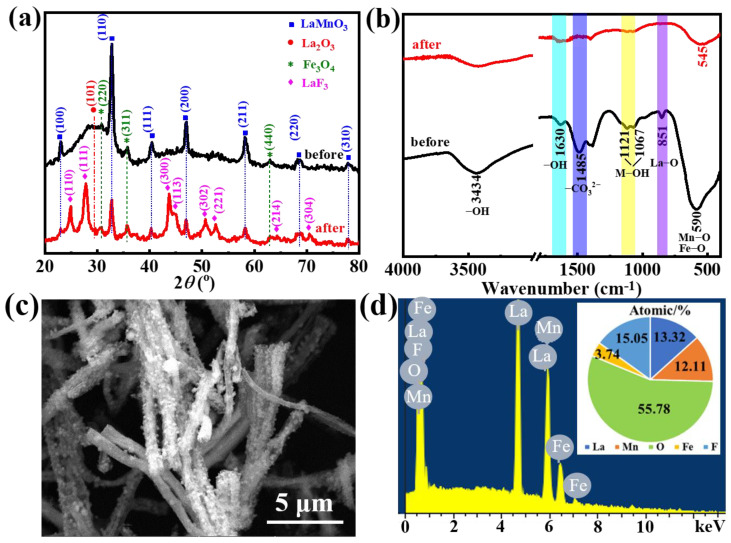
PXRD patterns (**a**) and FTIR spectra (**b**) of LMF11 NFs before and after F^−^ loaded, SEM image (**c**) and EDS (**d**) of LMF11 NFs after F^−^ uptake.

The defluoridation mechanism of LMF11 NFs was analyzed by PXRD and FTIR. Figure 12a shows the PXRD spectra of the LMF11 NFs and F^−^ adsorbed LMF11 NFs. Seven characteristic peaks appearing at 2θ = 23.0°, 32.8°, 40.5°, 47.1°, 58.2°, 68.6° and 77.8°, assigned to peak indices of (100), (110), (111), (200), (211), (220) and (310), respectively, portray the cubic perovskite phase of LaMnO_3_ (JCPDS NO. 74-0440) [33,77]. The detected characteristic peaks occur at 2θ = 30.7° (220), 35.8° (311) and 62.9° (440), signifying that the cubic phase of Fe_3_O_4_ has been generated [44]. Meanwhile, the wide peak located at 2θ = 29.5° (220) [78] assigns to La_2_O_3_. In summary, the LMF11 NFs are composed of La_2_O_3_, LaMnO_3_ and Fe_3_O_4_. After defluoridation, the disappearance of the diffraction peak ascribed to La_2_O_3_ and the presence of new reflections belonged to the hexagonal structure of LaF_3_ (PDF No.32-0483) [43] state that the ion exchange between -OH on the surface of LMF11 NFs and F^−^ is the dominating defluoridation mechanism of hydrous La_2_O_3_ in adsorption system. Moreover, the reflection intensity of Fe_3_O_4_ and LaMnO_3_ turns slightly weak after fluoride uptake owing to the decrease in crystallinity caused by the entry of F^−^ into the crystal lattice. 

As seen from the FTIR spectrum of the neat LMF11 NFs (Figure 12b), the bands at 3434, 1630, 1485, 851 and 590 cm^−1^ assign to the surface hydroxyl groups stretching mode, chemisorbed water bending mode, CO_3_^2−^ stretching mode, La-O stretching mode, and Mn-O along with Fe-O stretching mode [44,77,79]. Meanwhile, the two peaks ascribed to the metal hydroxyl bond (M-OH) [67,80] at 1121 and 1067 cm^−1^ are detected. After fluoride uptake, the bands at 3430 and 1632 cm^−1^ turn obviously weak and two peaks at 1127 and 1067 cm^−1^ are not detected, validating that the bonding of fluoride through electrostatic interaction as well as the replacement of -OH by F^−^ via ion exchange [81]. Moreover, the La-O band vanishes and the M-O bands (i.e., Mn-O and Fe-O) significantly change, revealing that metal-oxygen bands (La-O, Mn-O and Fe-O) are participated in defluoridation process [82].

F^−^ loaded LMF11 adsorbent was further verified by SEM and EDS (Figure 12c,d). The used LMF11 NFs have preserved fibrous morphology. However, the morphological surface of the used LMF11 NFs acquires modified, since there are lots of nanoparticles on the surface of F^−^ loaded LMF11 NFs after fluoride adsorption, indicating that fluoride ions have covered the LMF11 NFs surface and LaF_3_ nanoparticles have been yielded via ion exchange during the defluoridation process, which is consistent with PXRD analysis. Apparently, the EDS spectrum of F^−^ loaded LMF11 NFs portrayed in Figure 12d suggests the presence of La (13.32%), Mn (12.11%), F (15.05%), Fe (3.74%) and O (55.78%) in the used LCF11 NFs.

**Figure 13:**
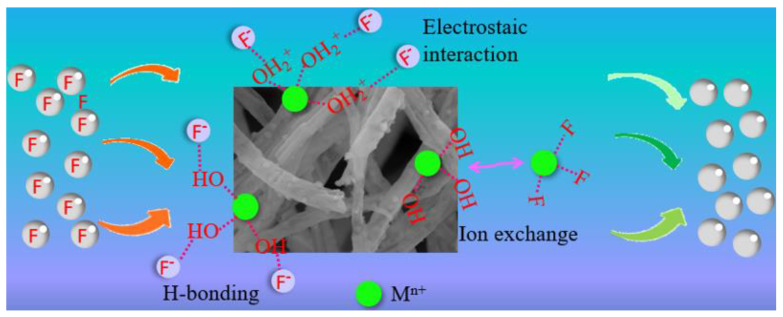
Possible F^−^ adsorption mechanism of LMF11 NFs.

The pH and pH_pzc_ investigation of LMF11 NFs on F^−^ removal reveal that it is more prone to higher removal efficiency in the acidic condition, owing to opposite charges on LMF11 NFs surface and F^−^ which is responsible for columbic attractions. The values of *E*_DR_ calculated by D-R isotherm are 14.06, 14.91, 16.58 and 17.96 kJ mol^−1^, respectively, at 15, 25, 35 and 45 °C, confirming that the F^−^ adsorption is owing to both ion exchange and chemisorption [29,83]. In a word, the defluoridation process onto LMF11 NFs is significantly governed by hydrogen bond, columbic interaction, as well as ion exchange. The plausible defluoridation mechanism by LMF11 NFs is depicted in Figure 13.

## 4. Conclusions

In this study, magnetic electrospun La-Mn-Fe tri-metal oxide nanofibers (LMF NFs) were developed through electrospinning and heat treatment and used for fluoride recovery. The prepared LMF11 NFs with a La:Mn molar ratio of 1:1 gained the highest fluoride adsorption amount. The results of the investigation were summarized as follows:

(1) The LMF11 NFs presented fiber morphology with an average diameter of 626 ± 57 nm. The results showed that the spherical Fe_3_O_4_ NPs were uniformly dispersed along the fiber axis without obvious agglomeration, and the total pore volume and surface area of LMF11 NFs were 0.276 cm^3^ g^−1^ and 55.81 m^2^ g^−1^, respectively. The isoelectric point (pH_pzc_) of LMF11 NFs was 6.47.
(2) The responsible factors such as initial pH values, shaking time, interfering ions, temperature, initial concentration of adsorbate and adsorbent for fluoride retention on LMF11 NFs were systematically conducted. The maximum adsorption amount of LMF11 NFs achieved at a pH of 3. With ascending LMF11 NFs dosage, the adsorption uptake descended from 188.21 to 82.77 mg F^−^/g, and the adsorption amount of LMF11 NFs increased with the ascend of initial fluoride and then reached equilibrium. The fluoride adsorption by the LMF11 NFs does not affect by the interfering anions, such as NO_3_^−^, Cl^−^, SO_4_^2−^ and CO_3_^2−^, with the exception of PO_4_^3−^ anions.
(3) The Langmuir isotherm and PSO model were favored divulging that the F^−^ adsorption on LMF11 NFs was seen as a single-layer chemisorption. The maximum binding amount calculated from the Langmuir isotherm model was as high as 173.30–199.60 mg F^−^/g at pH = 3 at 15–45 °C. A thermodynamic study proved that the defluoridation by LMF11 NFs is a spontaneous, endothermic as well as entropy increase process. The regeneration of the F^−^ loaded LMF11 NFs exhibited a high defluoridation efficiency of 94.76 mg F^−^/g at *C*_0_ = 20 mg F^−^/L up to three repetitions.
(4) On the basis of the isoelectric point, FTIR and PXRD analysis, it was confirmed that LMF11 NFs worked with the defluoridation mechanisms including hydrogen bonding, electrostatic attraction and ion exchange.

In summary, the fabricated LMF11 NFs possessed good defluoridation performance and high selective adsorption ability, indicating that the material has great potential as a promising adsorbent for fluoride decontamination. Further study will be conducted on the removal of phosphate, arsenate and other pollutants in the future. 

## Figures and Tables

**Table 1:** Four classical isotherms constants for defluoridation on LMF11 NFs at 15 to 45 °C.

Models	Parameters	*T* (°C)			
		15	25	35	45
Langmuir	*Q*_m_ (mg g^−1^)	173.30	189.03	193.05	199.60
	*K*_L_ (L mg^−1^)	1.7445	2.2383	2.4904	2.5825
	*R* ^2^	0.9997	0.9990	0.9987	0.9976
	*R*_L_	0.0095–0.0542	0.0073–0.0427	0.0066–0.0386	0.0064–0.0373
Freundlich	*K*_F_ (L g^−1^)	93.54	104.01	114.66	123.20
	1/*n*	0.2543	0.2460	0.2117	0.1961
	*R* ^2^	0.7863	0.7252	0.5627	0.4791
D–R	*k*_ad_’ × 10^2^ (mol^2^ kJ^−2^)	0.2530	0.2250	0.1820	0.1550
	*E* (kJ mol^−1^)	14.06	14.91	16.58	17.96
	*R* ^2^	0.8402	0.7725	0.5981	0.4976
Temkin	*K*_T_ (L g^−1^)	44.39	61.11	158.11	261.92
	*B*_T_ (J mol^−1^)	89.64	90.26	104.64	110.95
	*R* ^2^	0.8708	0.8358	0.6854	0.6161

**Table 3:** Kinetic constants for defluoridation on LMF11 NFs.

Models	Parameters	*C*_0_ (mg/L)	
		20	50
PFO	*k*_1_ × 10^2^ (min^−1^)	4.2040	5.3240
	*Q*_e_ (mg g^−1^)	7.04	38.03
	*R* ^2^	0.7212	0.9019
PSO	*k*_2_ × 10^2^ (g mg^−1^ min^−1^)	3.7012	0.5236
	*Q*_e, cal_ (mg g^−1^)	98.23	184.50
	*Q*_e, exp_ (mg g^−1^)	97.76	181.49
	*R* ^2^	0.9999	0.9999
Elovich	*α* (mg g^−1^ min^−1^)	1.8793 × 10^10^	1.6323 × 10^5^
	*β* (g mg^−1^)	0.2642	0.0725
	*R* ^2^	0.8714	0.9698
Weber–Morris	*k*_int1_ (mg g^−1^ min^−0.5^)	9.4357	14.4288
	*R* ^2^	0.9974	0.9954
	*C*	71.97	116.48
	*k*_int2_ (mg g^−1^ min^−0.5^)	1.1282	11.77
	*R* ^2^	0.9207	0.9988
	*C*	92.02	112.98
	*k*_int3_ (mg g^−1^ min^−0.5^)	0.2503	1.4632
	*R* ^2^	0.5567	0.8091
	*C*	95.94	170.48

**Table 4:** Thermodynamic factors of defluoridation by LMF11 NFs.

*T* (°C)	Δ*G*^0^ (kJ mol^−1^)	ln*K*_D_	Δ*H*^0^ (kJ mol^−1^)	Δ*S*^0^ (J mol^−1^ K^−1^)
15	−5.89	2.46	10.45	69.12
25	−6.69	2.70		
35	−7.08	2.76		
45	−7.64	2.89		

## Data Availability

The data are included within the article.

## Supplementary Materials

The following supporting information can be downloaded at: https://www.mdpi.com/article/10.3390/polym14245417/s1, Table S1. Factors and their levels; Table S2. The design of experiment for the adsorption of fluoride and the actual and predicted responses; Table S3. ANOVA results for adsorption capacity; Figure S1. (a) Normal probability plot; (b) Comparison between actual and predicted adsorption efficiency; Figure S2. 3D surface model graphs of fluoride adsorption. Refs. [55,56,84,85,86].
