# Supplementary material for: Template-Free Synthesis of Magnetic La-Mn-Fe Tri-Metal Oxide Nanofibers for Efficient Fluoride Remediation: Kinetics, Isotherms, Thermodynamics and Reusability
Source: Polymers (Basel). 2022 Dec 11;14(24):5417. doi: 10.3390/polym14245417 (PMC9784745; doi:10.3390/polym14245417)
Supplement: Supplementary file 1 [file polymers-14-05417-s001.zip › polymers-2064616-supplementary v2.pdf]

Supporting Information

Template-Free Synthesis of Magnetic La-Mn-Fe Tri-Metal Oxide Nanofibers for Efficient Fluoride Remediation: Kinetics, Isotherms, Thermodynamics and Reusability

Shaoju Jian [1], Yuhuang Chen [1], Fengshuo Shi [1], Yifei Liu [1], Wenlong Jiang [1], Jiapeng Hu [1,*], Xiaoshuai Han [1,2], Shaohua Jiang [1,2,*] and Weisen Yang [1,*]

Removal efficiency (%): $\eta = \frac{C_0 - C_e}{C_0} \times 100\%$

Langmuir model: $\frac{C_e}{Q_e} = \frac{C_e}{Q_m} + \frac{1}{bQ_m}$ (1)

dimensionless constant separation factor: $R_L = \frac{1}{1 + K_L C_0}$ (2)

Freundlich model: $\ln Q_e = \ln k_F + \frac{1}{n} \ln C_e$ (3)

Dubinin-Radushkevich (D-R) model: $\ln Q_e = \ln Q_m - \beta R^2 T^2 \ln^2(1 + \frac{1}{C_e})$ (4)

$E = \frac{1}{\sqrt{2\beta}}$ (5)

Temkin model: $Q_e = \frac{RT}{B_T} \ln K_T + \frac{RT}{B_T} \ln C_e$ (6)

PFO model: $\ln(Q_e - Q_t) = \ln Q_e - k_1 t$ (7)

PSO model: $\frac{t}{Q_t} = \frac{1}{k_2 Q_e^2} + \frac{t}{Q_e}$ (8)

Elovich model: $Q_t = \frac{1}{B} \ln(\alpha B) + \frac{1}{B} \ln t$ (9)

Weber-Morris model: $Q_t = K_{int} t^{\frac{1}{2}} + C$ (10)

Abbreviations

C_0 original concentration of F^- (mg L^{-1})

C_e equilibrium concentration of F^- (mg L^{-1}), while (mol L^{-1}) in D-R model

η removal efficiency (%)

m	amount of LMF11 NFs (g)
Q_e	adsorption capacity of F^- at equilibrium (mg g^{-1}), while (mol g^{-1}) in D-R model
Q_m	maximum adsorption capacity (mg g^{-1})
Q_t	adsorption capacity of F^- at any time t (mg g^{-1})
t	time (min)
K_L	constant of the Langmuir isotherm (L mg^{-1})
R_L	dimensionless constant separation factor
K_F	Freundlich adsorption equilibrium constant (L mg^{-1})
n	Freundlich linear index
β	a D-R model constant associated with adsorption energy
K_T	Temkin isothermal equilibrium binding constant (L/mg)
B_T	Temkin isothermal constant (J/mol)
E	free energy (kJ mol^{-1})
PFO	pseudo-first order
PSO	pseudo-second order
k_1	the rate constant of PFO (min^{-1})
k_2	the rate constant of PSO (g $mg^{-1} \cdot min^{-1}$)
α	the rate of initial sorption (mg $g^{-1} \cdot min^{-1}$)
B	desorption constant (g/mg)
k_{int}	constant of intra-particle diffusion (g mg^{-1} $min^{-0.5}$)
C	the thickness of boundary layer
ΔG	the Gibbs free energy change (kJ mol^{-1})
ΔH	the enthalpy change (kJ mol^{-1})
ΔS	the entropy change (J mol K^{-1})
R	the ideal gas constant (8.314 J mol K^{-1})
T	the absolute temperature (K)

1. Optimization of adsorption conditions

1.1 Design of experiment

In this work, the effect of independent variables was investigated response surface methodology (RSM)[1], and the experimental design for the optimization of the fluoride removal conditions was carried out using Design expert software viz., the BBD (Box-Behnken Design). Based on the aforementioned analysis, the three factors of pH (*A*), initial concentration of F^- (*B*), and LMF11 NFs dosage (*C*) were designed, and three levels of each factor were set and fitted to the model to determine the optimum reaction conditions. The levels of parameter were given in Table S1. A total of 17 samples were obtained and analyzed (Table S2). The significance of the model was evaluated using analysis of variance (ANOVA) results with a confidence level of 95%. The experimental data were fitted using a multinomial quadratic regression model.

Table S1 Factors and their levels.

Factors	Code	Code variable levels		
		-1	0	1
pH	A	2	3	4
LMF11 NFs dosage (g)	B	0.01	0.015	0.02
Initial Concentration (mg/L)	C	40	50	60

Table S2 The design of experiment for the adsorption of fluoride and the actual and predicted responses.

Run	Factors			Responses	
	A	*B*	*C*	Actual	Predicted
1	4	0.02	50	90.44	88.83
2	3	0.02	60	80.48	82.54
3	2	0.015	60	62.46	63.09
4	3	0.01	40	83.95	81.89
5	4	0.01	50	62.21	64.09
6	3	0.015	50	90.41	91.44
7	3	0.02	40	94.22	96.46
8	3	0.015	50	90.77	91.44
9	2	0.01	50	64.16	65.77
10	3	0.015	50	90.01	91.44
11	3	0.015	50	92.48	91.44
12	2	0.02	50	91.32	88.63
13	3	0.015	50	93.53	91.44
14	3	0.01	60	52.55	50.31
15	4	0.015	40	86.13	85.50
16	4	0.015	60	65.63	65.18
17	2	0.015	40	87.80	88.25

2. Results and Discussion

2.1 Analysis of variance and significance test

Analysis of variance (ANOVA) is essential to determine the significance and adequacy of the model, and it is used to determine the model and experimental error. The significance of the quadratic model was indicated by F-value and P-value tests and the fair value of the squared correlation (R^2). The experimental results in Table S2 were subjected to significance tests and surface ANOVA, and the results are shown in Table S3. The fluoride removal efficiency (Y) is represented by the response surface quadratic polynomial for each of the three factors, pH (A), dosage (B), and initial concentration of F^- (C). The equation as following:

$$Y=91.44-0.1662*A+11.70*B-11.37*C+0.2675AB+1.21*AC+4...42*BC-8.35*A^2-6.06*B^2-7.58*C^2 \quad (1)$$

Where Y is removal efficiency, A is pH, B is the dosage of adsorbent, C is the initial concentration of fluoride.

Table S3 ANOVA results for adsorption capacity

Source	Sum of	df	Mean	F-value	P-value	significance
model	2983.42	9	331.49	48.00	<0.0001	**
A-pH	0.2211	1	0.2211	0.0320	0.8631	#
B-dosage	1094.89	1	1094.89	158.53	<0.0001	**
C-initial	1034.67	1	1034.67	149.81	<0.0001	**
AB	0.2862	1	0.2862	0.0414	0.8445	#
AC	5.86	1	5.86	0.8497	0.3878	#
BC	77.97	1	77.97	11.29	0.0121	**
A^2	293.66	1	293.66	42.52	0.0003	**
B^2	154.43	1	154.43	22.36	0.0021	**
C^2	242.16	1	242.16	35.06	0.0006	**
Residual	48.35	7	6.91			
Lack of Fit	39.34	3	13.11	5.83	0.0609	#
Pure Error	9.00	4	2.25			
Cor Total	3031.77	16				
R^2	0.9841					
Adjusted R^2	0.9636					
Adequate	22.8925					
C.V%	3.24%					

"**" represent the significant, "#" represent the not significant.

As can be seen from the Table S3, the F-value of the model is 48, indicating that the model is significant and has a good prediction of fluorine removal rate, there is only a 0.01% chance that an F-value this large could occur due to noise. P-value < 0.05 indicates that the model has 95% confidence and the model is reliable [2].

Adequate precision (AP) represents the signal-to-noise ratio and in general its value is >4. In this experiment, Adequate precision (AP) is equivalent to 22.89, which satisfies the requirement. The correlation coefficient (R^2) was 0.9841 (>0.9) and the C.V% was 3.24%, indicating that the data had a good fit to the quadratic model, that the model predictions were highly accurate and that the predictions were valid and valuable [3,4]. Significance tests of the model coefficients revealed that the effects of B, C, BC, A^2, B^2 and C^2 on removal rates were significant ($p < 0.05$). That are adsorbent dosage, initial concentration (F^-), interaction between dosing, and initial fluorine concentration, square of pH, square of dosing, and square of initial fluorine concentration had a more significant effect on removal efficiency, while other factors and their interactions did not have a significant effect on removal effect. Comparing the F-values, it can be observed that the order of the independent variables effect on fluoride removal (from the strongest to the weakest) is dosing, initial concentration of fluoride and pH, where dosing has the largest F-value, which is strongly correlated and has a highly significant influence; pH has the smallest F-value, which indicates a weak correlation in the experimental range and has a small influence on the response values. R^2 of 0.9841 indicates a great correlation between the actual adsorption effect and the predicted removal. This suggests that the model can be used to predict the performance of the material over the range of study. The points between residuals and probabilities were linearly distributed and the plot between residuals and probabilities (Figure S1a) verified the assumption of a well fit of the model predictions and a normal distribution of model errors. As shown in Figure S1b, the relationship between actual and predicted removal efficiency shows a linear relationship, and the points plotted are clustered around a straight line, indicating proper dispersion of the data and good fit of the model, which suggests that the model predictions are significant [1].

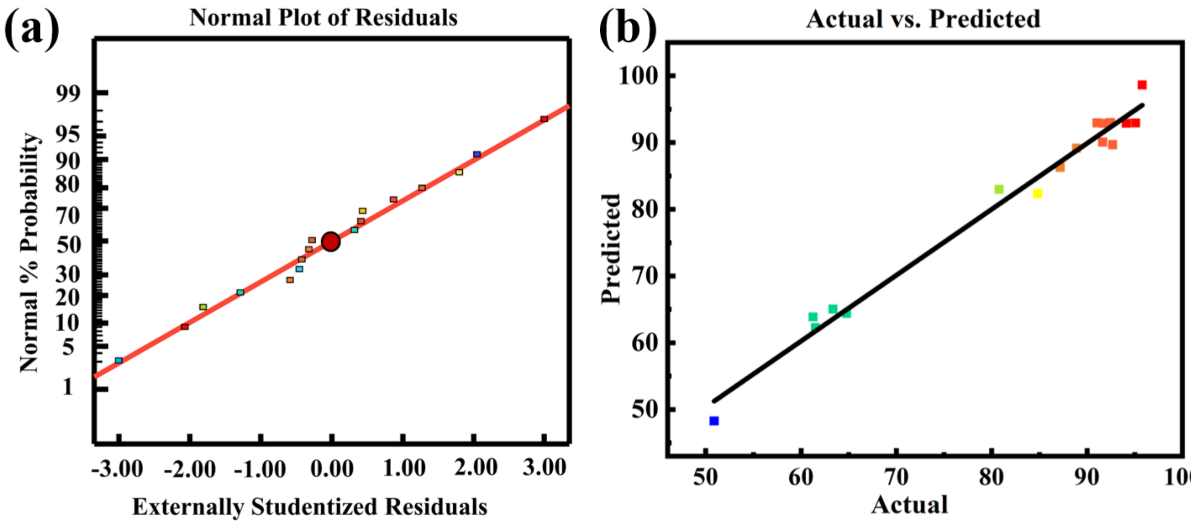

Figure S1. (a) Normal probability plot; (b) Comparison between actual and predicted adsorption efficiency

2.2 Interaction of each factor

The 3D response surface plots were carried out to analyze the effect of factor interactions on fluoride removal, and the results are shown in Figure S2a~c. The incline of 3D response surface is comparatively flat, demonstrating that the interaction between the factors is not obvious [5]. Figure S2a shows the effect of the interaction of AB (pH and adsorbent dosing amount), from which it can be seen that the removal efficiency gradually increases with increasing dosing amount, but the removal efficiency reaches a maximum at around pH levels 3, and continuing to increase pH has a slightly negative effect with a small downward trend. This indicates that the interaction of factor A and B has a small effect on the removal efficiency. Figure S2b shows a 3D surface of the effect of the interaction between AC (pH and initial fluoride concentration) on the adsorption effect, from which it can be watched that the adsorption removal rate changes less as pH increases, but the adsorption capacity changes more as the initial fluorine concentration increases, indicating that the initial concentration has a more significant effect on the removal efficiency than pH. And the Figure S2c shows the effect of BC (LMF11 NFs dosage and initial fluoride concentration) on the removal efficiency. It can be seen that the interaction of factor BC has the greatest effect on the removal efficiency, with the steepest 3D surface, and an increase in both initial concentration and dosing rate results in a sharp increase in removal effectiveness. The analysis of the interaction of the three factors shows that the effect of pH on the removal effect is small and the effect of both the initial concentration and LMF11 NFs dosage is more pronounced, which is consistent with the results shown in the ANOVA (Table S3).

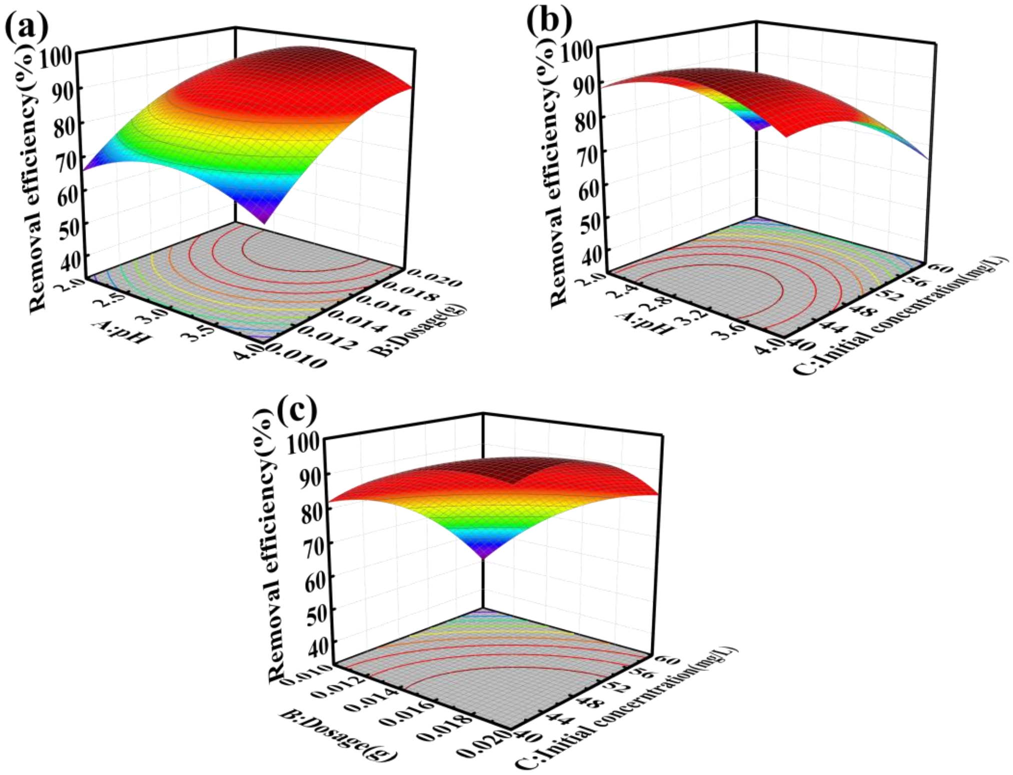

Figure S2. 3D surface model graphs of fluoride adsorption

2.3 Adsorption optimization and confirmation

Predicted maximum efficiency according to the numerical optimization results based on RSM is found to be 96.19% in the optimum value of variables, that is, 44.84 mg/L, 0.016 g and 2.77 for initial fluoride concentration, LMF11 NFs dosage, and pH, respectively. The experimental fluoride removal efficiency based on the optimum conditions obtained is reached to be 94.08% in triplicate experiments. Only a very small difference between the predicted and experimental results is observed.

References

1. Ghumman, A.S.M.; Shamsuddin, R.; Nasef, M.M.; Nisa Yahya, W.Z.; Abbasi, A. Optimization of synthesis of inverse vulcanized copolymers from rubber seed oil using response surface methodology. *Polymer* **2021**, *219*, 123553, doi:10.1016/j.polymer.2021.123553.
2. Siyal, A.A.; Shamsuddin, M.R.; Khahro, S.H.; Low, A.; Ayoub, M. Optimization of synthesis of geopolymer adsorbent for the effective removal of anionic surfactant from aqueous solution. *J. Environ. Chem. Eng.* **2021**, *9*, doi:10.1016/j.jece.2020.104949.
3. Nguyen, D.T.C.; Vo, D.-V.N.; Nguyen, T.T.; Nguyen, T.T.T.; Nguyen, L.T.T.; Tran, T.V. Optimization of

tetracycline adsorption onto zeolitic–imidazolate framework-based carbon using response surface methodology. *Surf. Interfaces.* **2022**, *28*, doi:10.1016/j.surfin.2021.101549.

4. Kalsido, A.W.; Meshesha, B.T.; Behailu, B.M.; Alemayehu, E. Optimization of Fluoride Adsorption on Acid Modified Bentonite Clay Using Fixed-Bed Column by Response Surface Method. *Molecules* **2021**, *26*, doi:10.3390/molecules26237112.

5. Yang, Y.; Li, X.; Gu, Y.; Lin, H.; Jie, B.; Zhang, Q.; Zhang, X. Adsorption property of fluoride in water by metal organic framework: Optimization of the process by response surface methodology technique. *Surf. Interfaces.* **2022**, *28*, 101649, doi:10.1016/j.surfin.2021.101649.
